# Association of Direct-Acting Antiviral Therapy for Hepatitis C With After-Treatment Costs Among Medicare Beneficiaries

**DOI:** 10.1001/jamanetworkopen.2020.8081

**Published:** 2020-06-30

**Authors:** Jeah Jung, Roger Feldman, Yamini Kalidindi, Thomas Riley

**Affiliations:** 1 Department of Health Policy and Administration, The Pennsylvania State University College of Health and Human Development, University Park; 2 Division of Health Policy and Management, University of Minnesota School of Public Health, Minneapolis; 3 Department of Medicine, The Pennsylvania State University College of Medicine, Hershey

## Abstract

**Importance:**

Direct-acting antiviral (DAA) therapy for hepatitis C is highly effective but expensive. Evidence is scarce on whether DAA therapy reduces downstream medical costs.

**Objective:**

To examine the association of DAA therapy with posttreatment medical costs among Medicare beneficiaries.

**Design, Setting, and Participants:**

This retrospective cohort study obtained data from various Medicare claims files for 2013 to 2017. The study population comprised patients with a hepatitis C diagnosis in 2014 who were enrolled in fee-for-service Medicare and Part D. Multivariate regression models were used to compare changes in medical costs over a 30-month posttreatment follow-up period between patients who used DAA therapy (treatment group) and a propensity score–matched cohort of patients who did not use DAA (control group). The model was estimated separately for patients with and those without cirrhosis. Data were analyzed between September 1, 2019, and March 31, 2020.

**Exposures:**

Completion of DAA therapy.

**Main Outcomes and Measures:**

Two outcomes were established: hepatitis C or liver disease–related costs and total medical costs. Costs were measured by Medicare-allowed payments, which included Medicare reimbursements, patient responsibilities, and third-party payments.

**Results:**

A propensity score–matched cohort of 15 198 patients (9038 men [59.5%]; mean [SD] age, 60.2 [10.8] years) was analyzed. During the first 6 months after DAA therapy, hepatitis C or liver disease–related costs decreased by $2498 (95% CI, −$3356 to −$1640) in patients with cirrhosis and by $486 (95% CI, −$603 to −$369) in patients without cirrhosis compared with control or untreated patients. Cumulative reductions in hepatitis C or liver disease–related costs during 30 months after DAA treatment were $15 808 (95% CI, −$22 530 to −$9085) in patients with cirrhosis and $5372 (95% CI, −$6384 to −$4360) in patients without cirrhosis. Among those who used DAA therapy compared with control patients, total medical costs decreased by $2905 (95% CI, −$4832 to −$979) in patients with cirrhosis and by $1287 (95% CI, −$2393 to −$283) in patients without cirrhosis during the first 6 months after DAA therapy. No statistically significant association was found between DAA therapy and total medical cost reductions after 12 months of follow-up. Cumulative reductions in total costs during 30 months after DAA therapy were $7074 (95% CI, −$18 448 to $4298) in patients with cirrhosis and $7497 (95% CI, −$14 287 to −$709) in patients without cirrhosis.

**Conclusions and Relevance:**

This study reported that DAA therapy appeared to be associated with a decrease in hepatitis C or liver disease–related costs for 30 months after treatment and with reduction in total medical costs for only 12 months after treatment in patients with or without cirrhosis. Longer-term follow-up studies with diverse outcomes are necessary to assess the value of DAA therapy.

## Key Points

**Question:**

Is direct-acting antiviral therapy for hepatitis C associated with a reduction in posttreatment medical costs among patients with Medicare coverage?

**Findings:**

In this cohort study of 15 198 patients with hepatitis C, direct-acting antiviral therapy was associated with a considerable reduction in hepatitis C or liver disease–related costs for 30 months after treatment, but it was associated with a substantial decrease in total medical costs for only 12 months after treatment.

**Meaning:**

These findings suggest that the costs of other conditions eventually outweigh the reduction in hepatitis C or liver disease–related costs in patients who use direct-acting antiviral drugs but still need medical care after hepatitis C infection has been cured; studies with longer-term follow-up and diverse outcomes are necessary to ascertain the value of this treatment.

## Teaser

This cohort study analyzes the cost implications for Medicare of direct-acting antiviral medications used by beneficiaries with or without hepatitis C–related liver disease.

## Introduction

Hepatitis C is an important public health problem in the United States for several reasons. It is a leading cause of serious and costly liver diseases, such as cirrhosis and liver cancer.^1^ The financial burden it imposes is estimated to exceed $10 billion annually.^2^ Hepatitis C was associated with more deaths than any other infectious disease in 2014 and 2015.^3,4^

The introduction of highly effective direct-acting antiviral (DAA) therapy has provided an unprecedented opportunity to address hepatitis C.^5,6,7,8^ However, high prices of DAA drugs have led third-party payers to restrict coverage for these medications while demanding more information on cost implications and the value of new treatments.^9,10,11,12^ Although the prices of DAA drugs have decreased as more new drugs have entered the market, they are still high, and coverage by some payers remains restrictive.^12,13^

Previous studies have evaluated the cost-effectiveness of DAA therapy compared with traditional peginterferon therapy and generally reported that DAA drugs would be an optimal treatment despite their high prices because they could reduce downstream medical costs and/or extend lives with their high cure rates.^14,15,16,17^ However, in calculating posttreatment medical costs, those studies considered only the estimated costs associated with the expected hepatitis C progression (eg, costs of cirrhosis and hepatocellular carcinoma) probably because they lacked data on actual posttherapy total medical costs.^14,18,19^ Patients with hepatitis C infection have several comorbidities because of the extrahepatic manifestation of hepatitis C,^20,21,22,23^ and patients need medical care to manage those conditions even after the infection is cured. Information on total medical costs is crucial for assessing the cost-effectiveness or value of a new treatment, but evidence on actual cost implications after DAA therapy is scarce.

A few recent studies examined total medical costs 1 year after DAA therapy using data from patients with Medicaid coverage or private insurance.^24,25^ One study found that medical costs 1 year after DAA treatment decreased from $14 014 to $12 327 among Medicaid beneficiaries^24^; however, this work did not have a control or untreated group. Another study of commercial enrollees with cirrhosis found that total medical cost 1 year after DAA treatment was $19 300 compared with $33 039 in the untreated group; however, that study did not account for the difference in pretreatment costs, which were $17 537 in patients who used DAA therapy and $23 798 in those who did not use it.^25^

To our knowledge, no research has been conducted to analyze the follow-up costs more than 1 year after DAA treatment. In addition, no study has examined the cost implications for Medicare, in which many baby boomers (the group with the highest prevalence of hepatitis C) are enrolled.^1,26^ Medicare also covers younger adults with disabilities and low income, the other group with a high prevalence of hepatitis C.^9^ Medicare is thus among the largest payers of DAA therapy.^27,28^ In the present study, we examined how DAA treatment was associated with medical costs by following up patients with Medicare for up to 30 months after they completed DAA treatment. The goal of this study was to offer the first evidence of DAA cost implications for Medicare beyond a 1-year follow-up.

## Methods

This cohort study was approved by the Pennsylvania State University Institutional Review Board, which granted a waiver of informed consent because the study used exclusively retrospective administrative records. We followed the Strengthening the Reporting of Observational Studies in Epidemiology (STROBE) reporting guideline. Data were analyzed between September 1, 2019, and March 31, 2020.

### Data Sources

The primary data were obtained from Medicare claims files for 2013 to 2017. To identify medical care use and costs, we analyzed data for these claim types: inpatient, skilled nursing facility, hospital outpatient, and carrier (noninstitutional, such as physician office) services. The 2013 files were used only to ensure no hepatitis C claims were made before 2014. We analyzed Medicare Part D data to identify DAA therapy use and costs. Master Beneficiary Summary Files provided data on demographics and health risk factors for each beneficiary.

### Study Population

The study population comprised Medicare beneficiaries who had a recent (in 2014) diagnosis of hepatitis C after a 1-year washout period. We identified patients with hepatitis C using the standard algorithm of the Centers for Medicare and Medicaid Services (details in eAppendix 1 in the Supplement).^29^ In addition, patients had to be enrolled in fee-for-service Medicare (ie, 0 months in Part C) and Part D during the study period (January 1, 2014, to December 31, 2017).

**Figure 1.**
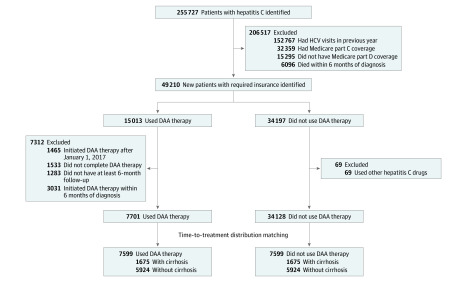
Study Sample Selection DAA indicates direct-acting antiviral; HCV, hepatitis C virus.

From the total population, we selected the study sample as shown in Figure 1. Patients who died within 6 months after diagnosis were excluded; DAA drugs were unlikely to be prescribed for those patients because a course of DAA therapy lasted 3 to 6 months. We identified the treatment group as patients who used 1 of the following DAA drugs: elbasvir and grazoprevir; ledipasvir and sofosbuvir; and ombitasvir, paritaprevir, and ritonavir plus dasabuvir, sofosbuvir, or sofosbuvir and velpatasvir. We assigned an index date to each patient as the first date of DAA prescription fills. Patients with an index date on or after January 1, 2017, were excluded to allow all patients to have 6 months of DAA therapy and at least one 6-month follow-up period after treatment. We included patients who completed DAA therapy, which was defined as filling prescriptions for the expected duration of the drug, as identified in package inserts or randomized clinical trials (eAppendix 2 in the Supplement). We excluded patients with an index date within 6 months after diagnosis because those patients did not have pretreatment costs related to hepatitis C or liver disease—a key study variable.

The comparison (control) group comprised patients who did not use DAA therapy during the study period. Those who were treated with non-DAA hepatitis C treatments were excluded (N = 69; 0.02%). We selected comparison patients using time-to-treatment distribution matching.^30^ We assigned a hypothetical treatment date to each DAA-untreated patient to make the distribution of time from diagnosis to treatment the same as that of the treatment group. Patients in the control group had to have 6 months before the assigned hypothetical treatment date and at least one 6-month follow-up after the hypothetical treatment.

We used propensity score matching to balance patient characteristics (demographics and health risks) during the treatment period and pretreatment spending. We matched patients with cirrhosis and those without cirrhosis separately. Matching in each group was performed with patients who had the same time-to-treatment range. The definitions and data sources of all study covariates are included in eTable 1 in the Supplement, and eTables 2 and 3 in the Supplement present results from the logistic regression to calculate propensity scores.

### Follow-up Periods

We defined the pretreatment period as 6 months before the index date (eFigure in the Supplement). The treatment period was the first 6 months after the index date. To assess medical costs after the completion of DAA therapy, we excluded records from the treatment period. We followed up patients up to 30 months after the treatment period. We required that all patients have follow-up in the first 6 months after treatment. Subsequent follow-up periods included only patients who were alive in each period to incur medical costs over the full 6 months. To perform a sensitivity analysis, we created a separate sample of patients who were alive throughout the study period.

### Outcomes

We constructed 2 outcomes: hepatitis C or liver disease–related medical costs and total medical costs at 6-month intervals from the index date. We measured costs by Medicare-allowed payments, which included Medicare reimbursements, patient responsibilities, and any third-party payments (eTable 1 in the Supplement). We identified costs of inpatient, skilled nursing facility, emergency department, outpatient, and carrier services, but we did not include outpatient drug costs. All costs were measured in 2017 dollars, using the Consumer Price Index to adjust for inflation.

Total DAA costs were calculated as the sum of payer and patient responsibilities to enable their comparison with follow-up medical costs. The drug costs reported in the Medicare Part D data do not include manufacturer rebates or discounts. However, those rebates are accounted for in the Medicare payments to Part D plans. Thus, we discounted total DAA costs by 22%, the average discount on total drug costs for all brand-name Part D drugs in 2016.^31^

### Statistical Analysis

We used multivariate regression in a propensity score–matched cohort at the person-period level. The regression model controlled for patient characteristics, which may change over follow-up periods after matching. We compared posttreatment changes in medical costs between the treatment group (used DAA therapy) and control group (did not use DAA therapy), a difference-in-differences approach. eAppendix 3 in the Supplement provides details of the regression and describes the variables used in the model. Among those variables were indicators that controlled for a common time trend over the follow-up periods and an indicator of DAA therapy use that controlled for permanent differences between the treatment and control groups. Interaction terms between the time trend indicators and the DAA therapy use indicator were the variables of interest. Their coefficients measured the differences in changes in medical costs over time between the 2 groups. The sum of these coefficients represented the cumulative cost change during the study period.

We estimated a generalized linear model with a log link and the gamma distribution to account for the skewed distribution of the cost variables.^32,33^ We estimated the model separately for patients with and those without cirrhosis. This separate analysis approach allowed heterogeneity in the associations of all covariates with follow-up costs between the 2 groups. We clustered SEs at the patient level.

A 2-tailed *P* < .05 was considered statistically significant. We compared patient characteristics between the treatment and control groups using unpaired, 2-tailed *t* tests for binary and continuous variables and with χ^2^ tests for categorical variables. We used SAS (version 9.4; SAS Institute Inc) and Stata (version 15; StataCorp LLC) for the analysis.

### Additional Analyses

First, we performed a sensitivity analysis with patients who were alive throughout the study period to account for potential differences in mortality between the treatment and control groups. Second, we examined the association of DAA use with follow-up costs by time from hepatitis C diagnosis to treatment. We grouped patients who used DAA therapy into 2 categories of time to DAA therapy initiation: within 6 to 12 months and after 12 months of diagnosis. We compared changes in medical costs between patients in each of the 2 categories and patients in the control group. The model controlled for common time trends, permanent differences between patients with early and delayed treatments, and patient characteristics.

## Results

### Descriptive Results

The primary analysis included a propensity score–matched cohort of 15 198 patients (9038 men [59.5%]; mean [SD] age, 60.2 [10.8] years). Characteristics of patients with or without cirrhosis were similar between those who used DAA therapy (treatment group) and those who did not in the matched sample (control group) (Table 1). For example, the number of patients with cirrhosis was 1192 (71.2%) in the treatment group and 1157 (69.1%) in the control group (*P* = .41). Patients who were not matched to the treatment cohort exhibited characteristics that were different from the characteristics of those who were matched (eTable 4 in the Supplement). For example, the number of patients with cirrhosis was 1350 of 2844 unmatched patients (47.5%) in the treatment group and 1157 of 1675 matched patients (69.1%) in the control group (*P* < .001).

**Table 1.** Patient Characteristics in the Propensity Score–Matched Sample^a^

Variable	Patients with cirrhosis (n = 3350)			Patients without cirrhosis (n = 11 848)		
	Treatment group, No. (%) (n = 1675)	Control group, No. (%) (n = 1675)	*P* value^b^	Treatment group, No. (%) (n = 5924)	Control group, No. (%) (n = 5924)	*P* value^b^
Age, mean (SD), y	62.1 (10.2)	62.8 (8.8)	.03	59.4 (11.7)	59.7 (10.4)	.08
Age group, y						
<65	963 (57.5)	972 (58.0)		3827 (64.6)	3714 (62.7)	
65-70	381 (22.8)	364 (21.7)		1255 (21.2)	1319 (22.3)	
71-75	184 (11.0)	186 (11.1)		529 (8.9)	551 (9.3)	
>75	147 (8.8)	153 (9.1)	.91	313 (5.3)	340 (5.7)	.18
Sex						
Women	616 (36.8)	611 (36.5)		2463 (41.6)	2470 (41.7)	
Men	1059 (63.2)	1064 (63.5)	.86	3461 (58.4)	3454 (58.3)	
Race/ethnicity						
White	1193 (71.2)	1217 (72.7)		3959 (66.8)	3909 (66.0)	
Black	336 (20.1)	311 (18.6)		1583 (26.7)	1634 (27.6)	
Hispanic	61 (3.6)	61 (3.6)		159 (2.7)	164 (2.8)	
Other	85 (5.1)	86 (5.1)	.75	223 (3.8)	217 (3.7)	.73
Cirrhosis type						
Decompensated	483 (28.8)	518 (30.9)		NA	NA	
Compensated	1192 (71.2)	1157 (69.1)	.41	NA	NA	
Conditions						
HIV/AIDS	62 (3.7)	58 (3.5)	.71	457 (7.7)	466 (7.9)	.76
Hepatocellular cancer	111 (6.6)	137 (8.2)	.09	31 (0.5)	37 (0.6)	.47
Anemia	683 (40.8)	696 (41.6)	.65	1515 (25.6)	1624 (27.4)	.02
Lung disease	462 (27.6)	499 (29.8)	.16	1386 (23.4)	1511 (25.5)	.01
Cancer	217 (13.0)	207 (12.4)	.60	645 (10.9)	701 (11.8)	.11
Cardiac disease	1307 (78.0)	1324 (79.0)	.47	4052 (68.4)	4172 (70.4)	.02
Dementia	98 (5.8)	87 (5.2)	.41	276 (4.7)	272 (4.6)	.86
Psychiatric conditions	832 (49.7)	858 (51.2)	.37	3048 (51.4)	3124 (52.7)	.16
Diabetes	665 (39.7)	689 (41.1)	.40	1736 (29.3)	1817 (30.7)	.32
Eye disease	303 (18.1)	304 (18.2)	.96	992 (16.8)	1051 (17.7)	.15
Kidney disorders	545 (32.5)	557 (33.2)	.66	1312 (22.3)	1395 (23.6)	.07
Drug and alcohol-related disorder	889 (53.1)	946 (56.5)	.05	2950 (49.8)	2917 (49.2)	.54
Bone disease	684 (40.8)	664 (39.6)	.48	2423 (40.9)	2549 (43.0)	.02
ESKD	72 (4.3)	69 (4.1)	.80	227 (3.8)	242 (4.1)	.48

Abbreviations: ESKD, end-stage kidney disease; NA, not applicable.

^a^ Patient characteristics were measured at baseline (time of treatment).

^b^ *P* values were calculated with unpaired, 2-tailed *t* tests for binary and continuous variables and with χ^2^ tests for categorical variables.

**Figure 2.**
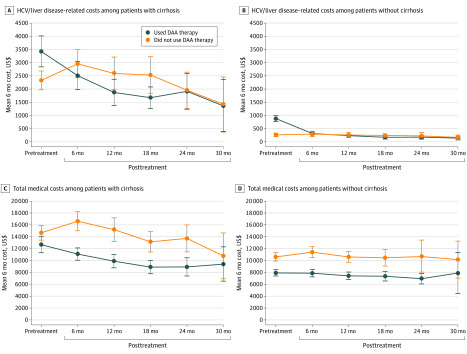
Unadjusted 6-Month Costs Over Time Between Patients Who Used and Patients Who Did Not Use Direct-Acting Antiviral (DAA) Therapy The costs are Medicare-allowed payments for inpatient, skilled nursing facility, emergency department, outpatient, and physician office visits. DAA therapy costs are not included. For those who did not use DAA therapy, posttreatment is a hypothetical period. eTable 3 in the Supplement provides the actual US dollar amounts. The bars indicate 95% CIs; HCV, hepatitis C virus.

Figure 2 plots unadjusted spending over time for patients with or without cirrhosis who used DAA therapy or who did not. All descriptive cost data are reported in eTable 5 in the Supplement. Among patients with cirrhosis, the mean 6-month hepatitis C or liver disease–related costs per patient who used DAA therapy decreased from $3422 before treatment to $2511 during the first 6 months after treatment (*P* < .001). Among patients without cirrhosis, the mean 6-month hepatitis C or liver disease–related costs per patient who used DAA therapy decreased from $879 before treatment to $311 after treatment (*P* < .001). Control patients showed a slight increase in hepatitis C or liver disease–related costs during the same period.

Among patients with cirrhosis, the mean 6-month total costs per patient who used DAA therapy decreased from $12 665 before treatment to $11 086 during 6 to 12 months after treatment (*P* < .001). Patients in the control group showed a slight increase in total costs during the 6- to 12-month follow-up period but a decrease from $15 200 to $13 135 (*P* < .001) during the 12- to 24-month follow-up period. Among patients without cirrhosis, the mean 6-month total costs per patient who used DAA therapy decreased from $7911 to $7881 (*P* < .001) during the 6- to 12-month follow-up period.

### Regression Results

Table 2 presents the difference-in-differences estimates for follow-up medical costs associated with use of DAA therapy. For patients with cirrhosis, hepatitis C or liver disease–related costs decreased by $2498 (95% CI, −$3356 to −$1640) during the first 6 months after treatment among those who used DAA therapy compared with nonusers and by $2350 (95% CI, −$3291 to −$1410) during the 6 to 12 months after treatment. For patients without cirrhosis, hepatitis C or liver disease–related costs decreased by $486 (95% CI, −$603 to −$369) during the first 6-month follow-up period among those who used DAA therapy compared with nonusers and by $607 (95% CI, −$735 to −$480) during the 6 to 12 months after treatment. This cost reduction lasted through the rest of the follow-up periods.

**Table 2.** Adjusted Changes in Outcomes Between Treatment and Control Groups, by Cirrhosis Status^a^

Variable	Patients with cirrhosis (n = 12 573)			Patients without cirrhosis (n = 46 498)		
	Changes in outcome (95% CI)		Differences (95% CI)	Changes in outcome (95% CI)		Differences (95% CI)
	Treatment group	Control group		Treatment group	Control group	
**Hepatitis C/liver disease–related costs, US$**						
Time after treatment, mo						
6	−3187 (−3896 to −2477)	−689 (−1256 to −122)	−2498 (−3356 to −1640)	−564 (−667 to −462)	−78 (−147 to −9)	−486 (−603 to −369)
12	−3041 (−3789 to −2293)	−691 (−1308 to −77)	−2350 (−3291 to −1410)	−710 (−836 to −584)	−103 (−169 to −36)	−607 (−735 to −480)
18	−2658 (−3357 to −1959)	−885 (−1706 to −64)	−1773 (−2740 to −805)	−659 (−777 to −541)	−102 (−182 to −22)	−557 (−675 to −439)
24	−1706 (−3041 to −371)	−1256 (−1814 to −698)	−450 (−1869 to 970)	−582 (−694 to −471)	−134 (−217 to −51)	−448 (−565 to −331)
30	−2809 (−3418 to −2200)	−1238 (−2464 to −12)	−1571 (−2895 to −247)	−513 (−610 to −415)	−136 (−235 to −39)	−376 (−499 to −253)
**Total medical costs, US$**						
Time after treatment, mo						
6	−3924 (−5101 to −2747)	−1019 (−2574 to 537)	−2905 (−4832 to −979)	−1082 (−1666 to −497)	206 (−685 to 1098)	−1287 (−2293 to −283)
12	−4079 (−5313 to −2845)	−1999 (−3625 to −373)	−2079 (−4054 to −105)	−1587 (−2194 to −981)	67 (−932 to 1067)	−1654 (−2689 to −620)
18	−3929 (−5271 to −2587)	−4172 (−5980 to −2365)	244 (−1911 to 2398)	−954 (−1673 to −236)	874 (−1335 to 3083)	−1828 (−4034 to 377)
24	−3209 (−5106 to −1311)	−2145 (−5189 to 899)	−1064 (−4595 to 2467)	−1733 (−2546 to −919)	−352 (−2160 to 1456)	−1380 (−3214 to 453)
30	−3135 (−6079 to −190)	−4500 (−8198 to −802)	1365 (−3347 to 6077)	−1631 (−3551 to 289)	−1012 (−3855 to 1832)	−619 (−4097 to 2859)

Abbreviation: DAA, direct-acting antiviral.

^a^ Multivariate regression was used to examine the association of DAA treatment with spending outcomes in a propensity score–matched cohort. Changes in medical costs were compared between patients who used and those who did not use DAA therapy over 30 months of posttreatment follow-up using a difference-in-differences approach. eAppendix 3 in the Supplement provides further details of the regression model and variables used in the model.

Among patients with cirrhosis, total medical costs decreased by $2905 (95% CI, −$4832 to −$979) during the first 6 months after treatment among those who used DAA therapy compared with nonusers and by $2079 (95% CI, −$4054 to −$105) during the 6 to 12 months after treatment. Total medical costs in patients without cirrhosis decreased by $1287 (95% CI, −$2293 to −$283) in the first 6 months after treatment among those who used DAA therapy compared with nonusers and by $1654 (95% CI, −$2689 to −$620) during the 6 to 12 months after treatment (Table 2). No cost reduction was statistically significant after that follow-up period.

Use of DAA therapy was associated with cumulative reductions in hepatitis C or liver disease–related costs of $15 808 (95% CI, −$22 530 to −$9085) in patients with cirrhosis and $5372 (95% CI, −$6384 to −$4360) in patients without cirrhosis during the 30 months of follow-up. In addition, use of DAA therapy was associated with cumulative reductions in posttreatment total medical costs of $7074 (95% CI, −$18 448 to $4298) in patients with cirrhosis and $7497 (95% CI, −$14 287 to −$709) in patients without cirrhosis.

### Drug Costs 

The mean (SD) DAA drug cost after 22% discounts were applied was $102 634 ($44 184) per patient with cirrhosis who used DAA therapy. The mean (SD) cost was $80 898 ($27 526) per patient without cirrhosis who used DAA therapy (eTable 5 in the Supplement).

### Additional Analyses

The sensitivity analysis with patients who were alive throughout the study period used a propensity score–matched cohort of 14 194 patients (8360 men [58.9%]; mean [SD] age, 60.0 [10.8] years). Results from this sensitivity analysis (eTable 6 in the Supplement) were similar to the primary analysis, confirming that differences in mortality between the treatment and control groups did not affect the study findings.

For the time-to-treatment analysis, the mean (SD) time from hepatitis C diagnosis to DAA initiation was 16 (7.2) months, with 2924 patients (38%) treated within 6 to 12 months and 4675 patients (62%) after 12 months of diagnosis. Patients with delayed treatment incurred lower hepatitis C or liver disease–related costs at the time of diagnosis ($1380) but higher hepatitis C or liver disease–related costs for 6 months before treatment ($2274) compared with those with early initiation of DAA therapy (eTable 7 in the Supplement).

**Figure 3.**
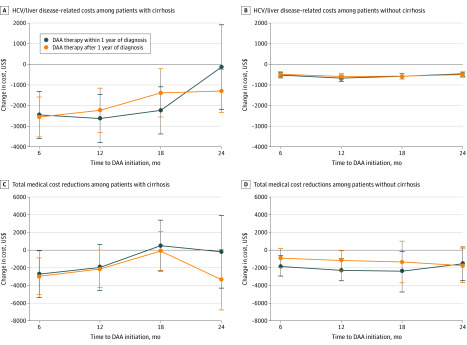
Adjusted Estimates of Cost Changes by Time to Direct-Acting Antiviral (DAA) Therapy Initiation Changes in medical costs are compared between those who initiated DAA therapy within 1 year of diagnosis and those who initiated 1 year after (see difference-in-differences approach described in eAppendix 3 in the Supplement). The model controls for common time trends and permanent differences across categories of DAA drugs. HCV indicates hepatitis C virus.

Time to DAA therapy initiation was associated with the degree of cost reduction (Figure 3 and eTable 8 in the Supplement). Among patients with cirrhosis, hepatitis C or liver disease–related costs during 6 to 12 months after treatment decreased by $2632 (95% CI, −$3800 to −$1465) if DAA therapy began within 6 to 12 months of diagnosis, but costs decreased by $2234 (95% CI, −$3306 to −$1163) if DAA therapy started after 12 months from diagnosis. Total costs among patients without cirrhosis decreased by −$1852 (95% CI, −$3086 to −$618) during the first 6 months if DAA therapy was initiated within 12 months of diagnosis but by −$905 (95% CI, −$1984 to $174) if treatment was delayed.

## Discussion

In analyzing claims data from Medicare patients with hepatitis C, we found that DAA therapy was associated with decreases in posttreatment hepatitis C or liver disease–related costs and total medical costs. The reduction in hepatitis C or liver disease–related costs lasted for 30 months after treatment, but the reduction in total medical costs lasted for only 12 months.

The short duration of the reduction in total medical costs is an important extension of previous work, which was limited to the finding based on 1-year follow-up.^24,25^ This finding suggests that the costs of other conditions eventually outweigh the reduction in hepatitis C or liver disease–related costs in patients who used DAA therapy and needed medical care after the hepatitis C infection was cured. Previous cost-effectiveness studies of DAA drugs lacked data on actual cost changes after DAA therapy and considered only estimated hepatitis C or liver disease–related costs.^14,18,19^ Findings of the present study suggest that total medical costs, including costs of other conditions, should be considered in assessing cost implications of DAA medications.

The estimate of 1-year reduction in hepatitis C or liver disease–related costs in the present study (approximately $4984) is less than the $13 739 cost reduction for commercial insurance enrollees reported in previous work.^25^ The reason for this difference may be that we compared changes in posttreatment costs between the treatment and control groups while controlling for differences in their pretreatment costs. The prior study compared costs in the posttreatment period only, even though the mean pretreatment costs in the treatment group were lower by $6261.^25^ Another explanation may be that Medicare covers older adults compared with commercial insurance. Thus, the health risks in the present sample may have been different from the health risks of commercial enrollees.

The association of DAA therapy with hepatitis C or liver disease–related cost reductions was larger in patients with cirrhosis than in patients without cirrhosis during the 30 months of follow-up. This finding suggests that DAA therapy helps mitigate the immediate aggravation of the hepatitis C condition in patients with advanced disease progression. This understanding seems consistent with the approach by payers to give treatment priority to patients with advanced fibrosis, particularly in the early years of DAA therapy availability.^12,34,35^ However, the present analysis was limited to 30 months of follow-up. Furthermore, this study found cost reductions among patients without cirrhosis, supporting the removal of DAA coverage restrictions by some payers.^36^ Access to DAA drugs is currently recommended for all persons with hepatitis C infection.^37^

Although previous cost-effectiveness studies have suggested that DAA drugs would be cost-saving,^14,17,18,19^ we found that cost-saving did not occur during the 30-month period after treatment. This seemingly inconsistent finding may be attributable to not assessing the value associated with improved quality of life and extended lives, which are key advantages of DAA therapy identified in past cost-effectiveness research.^14,17^ Studies of diverse outcomes over longer follow-up periods are necessary to assess the value of DAA drugs.

Initiating DAA therapy within 12 months after diagnosis was associated with the largest reduction in posttreatment hepatitis C or liver disease–related costs among patients with cirrhosis and total medical costs among patients without cirrhosis. Delayed treatment may have led to further hepatitis C progression and thus a small cost reduction. However, poor health at the time of diagnosis might have prevented patients from starting DAA therapy earlier. In either case, the finding suggests that time to DAA therapy initiation may be associated with the degree of cost reduction. Further exploration of the optimal time to treat may help increase the value of new DAA treatments.

### Limitations

This study has several limitations. First, the study did not use clinical information, such as viral load, genotype, and fibrosis stage, which are not recorded in Medicare claims. If those unobserved factors were associated with costs and their distribution was different in the treatment and control groups, selection bias (differences in patient characteristics between groups) may remain even with propensity score matching. Second, the study did not examine life-years saved after DAA therapy because analyzing mortality requires different approaches. However, the analysis with survivors suggested that the study results were not associated with mortality. Third, the data did not include DAA drugs that were introduced in or after 2017. New DAA drugs may have different cost implications compared with those included in the present study. Fourth, *International Classification of Diseases, Ninth Revision* codes transitioned to *International Statistical Classification of Diseases and Related Health Problems, Tenth Revision* codes during the study period. This transition might have affected the assessment of hepatitis C or liver disease–related costs. Fifth, the study analyzed data from fee-for-service Medicare patients for 30 months after treatment. The findings may not generalize to longer-term follow-up or to patients with coverage from Medicaid only, commercial insurance, or Medicare Advantage.

## Conclusions

In this cohort study, DAA therapy appeared to be associated with decreases in hepatitis C or liver disease–related costs for 30 months after treatment, with a larger decrease found among patients with cirrhosis than among patients without cirrhosis. The decrease in total medical costs lasted for only 12 months after DAA therapy in both patients with cirrhosis and without cirrhosis. Studies with longer-term follow-up periods and with diverse outcomes are necessary to assess the value of DAA therapy.

## Notes

Supplement. **eAppendix 1.** Diagnosis Codes Used to Identify Hepatitis C Virus (HCV) and HCV-Related Liver Disease **eAppendix 2.** Definition of the Completion of Treatment **eAppendix 3.** Details of Regression Specification **eFigure.** Index Date and Definition of Study Periods **eTable 1.** Definition and Data Sources of the Covariates and Outcomes Used in the Study **eTable 2.** Results From Logit Regression for Propensity Score (Cirrhosis Group) **eTable 3.** Results From Logit Regression for Propensity Score (Non-Cirrhosis Group) **eTable 4.** Patient Characteristics of Matched and Unmatched DAA Non-Users **eTable 5.** Unadjusted Spending Over Time and Death Rates Between DAA Users and Non-Users **eTable 6.** Results from Regression With Patients That Survived During Follow-Up **eTable 7.** Unadjusted Spending Among DAA Users by Time-to-Treatment **eTable 8. **Adjusted Outcomes Associated With DAA Use by Time to Treatment from HCV Diagnosis (by Cirrhosis)
