# Supplementary material for: Association of Direct-Acting Antiviral Therapy for Hepatitis C With After-Treatment Costs Among Medicare Beneficiaries
Source: JAMA Netw Open. 2020 Jun 30;3(6):e208081. doi: 10.1001/jamanetworkopen.2020.8081 (PMC7327546; doi:10.1001/jamanetworkopen.2020.8081)
Supplement: Supplement. — eAppendix 1. Diagnosis Codes Used to Identify Hepatitis C Virus (HCV) and HCV-Related Liver Disease eAppendix 2. Definition of the Completion of Treatment eAppendix 3. Details of Regression Specification eFigure. Index Date and Definition of Study Periods eTable 1. Definition and Data Sources of the Covariates and Outcomes Used in the Study eTable 2. Results From Logit Regression for Propensity Score (Cirrhosis Group) eTable 3. Results From Logit Regression for Propensity Score (Non-Cirrhosis Group) eTable 4. Patient Characteristics of Matched and Unmatched DAA Non-Users eTable 5. Unadjusted Spending Over Time and Death Rates Between DAA Users and Non-Users eTable 6. Results from Regression With Patients That Survived During Follow-Up eTable 7. Unadjusted Spending Among DAA Users by Time-to-Treatment eTable 8. Adjusted Outcomes Associated With DAA Use by Time to Treatment from HCV Diagnosis (by Cirrhosis) [file jamanetwopen-3-e208081-s001.pdf]

Supplementary Online Content

Jung J, Feldman R, Kalidindi Y, Riley T III. Association of direct-acting antiviral therapy for hepatitis C with after-treatment costs among Medicare beneficiaries. *JAMA Netw Open.* 2020;3(6): e208081. doi:10.1001/jamanetworkopen.2020.8081

eAppendix 1. Diagnosis Codes Used to Identify Hepatitis C Virus (HCV) and HCV-Related Liver Disease

eAppendix 2. Definition of the Completion of Treatment

eAppendix 3. Details of Regression Specification

eFigure. Index Date and Definition of Study Periods

eTable 1. Definition and Data Sources of the Covariates and Outcomes Used in the Study

eTable 2. Results From Logit Regression for Propensity Score (Cirrhosis Group)

eTable 3. Results From Logit Regression for Propensity Score (Non-Cirrhosis Group)

eTable 4. Patient Characteristics of Matched and Unmatched DAA Non-Users

eTable 5. Unadjusted Spending Over Time and Death Rates Between DAA Users and Non-Users

eTable 6. Results from Regression With Patients That Survived During Follow-Up

eTable 7. Unadjusted Spending Among DAA Users by Time-to-Treatment

eTable 8. Adjusted Outcomes Associated With DAA Use by Time to Treatment from HCV Diagnosis (by Cirrhosis)

This supplementary material has been provided by the authors to give readers additional information about their work.

©2020 Jung J et al. *JAMA Network Open*

eAppendix 1. Diagnosis Codes Used to Identify Hepatitis C Virus (HCV) and HCV-Related Liver Disease

Diagnosis codes
Chronic Hepatitis C Virus (HCV):
ICD-9-CM codes: 070.44, 070.54, 070.70, 070.71
ICD-10-CM codes: B18.2, B19.20, B19.21

HCV related liver disease:
ICD-9-CM codes: 571.5, 571.4, 571.41, 571.49, 571.8, 571.9, 573.1, 573.3, 573.9, 572.8, 572.4, V42.7, 456.2, 573.8, 155.0, 567.23, 782.4, 572.3, 572.2, 789.5, V02.69, V02.60, 707.9 ,70.6
ICD-10-CM codes: K74.0, K74.60, K74.69, K73.9, K73.0, K73.2, K83.8, K76.0, K76.89, K74.1, K76.9, K77, K716, K75.9, K76.9, K72.10, K72.90, K76.7, Z94.4, I85.0 ,K76.1, K76.89, C22.0, C22.7, C22.8, K65.2, R17, K76.6, K72.90, K72.91, R18.0, R18.8, B18.8, B18.9, L98.499 ,B19.0

Algorithm used to identify patients with hepatitis C virus

We identified patients with hepatitis C following the standard algorithm used by the Centers for Medicare and Medicaid Services (CMS): having at least 1 inpatient or skilled nursing facility claim; OR 2 hospital outpatient or carrier (physician service) claims of hepatitis C based on International Classification of Diagnosis (ICD) codes for HCV listed in the table above.

To identify newly diagnosed HCV patients, a one-year washout period was required – i.e., the patient should not have had any claim with HCV diagnosis in the prior year.

©2020 Jung J et al. *JAMA Network Open*

eAppendix 2. Definition of the Completion of Treatment

We used Medicare Prescription Drug Event (PDE) files to identify initiation of 1 of the following direct-acting antivirals (DAAs): elbasvir/grazoprevir, ledipasvir/sofosbuvir, ombitasvir/paritaprevir/ritonavir plus dasabuvir, sofosbuvir, and sofosbuvir/velpatasvir. The National drug codes or NDCs (a 11-digit unique numeric drug identifier created by Centers for Medicare and Medicaid Services) used to identify these drugs are listed below:

NDCs: 00006307401, 00006307402, 00074309328, 61958150101, 61958180101, 61958220101

All DAAs except sofosbuvir are administered as monotherapy or in combination with ribavirin. Sofosbuvir is concomitantly used with peginterferon, ribavirin, simeprevir, or daclatasvir.

DAA regimens vary by genotype and prior HCV treatment experience, which could not be identified from Medicare claims. However, the recommended duration of therapy indicated by package inserts is 12 weeks for most patients regardless of DAA regimen. For patients who have prior experience with other HCV treatments, the recommended treatment duration is 24 weeks (16 weeks for elbasvir/grazoprevir).

Results of recent randomized trials for patients without cirrhosis showed that 8-week treatments are effective for elbasvir/grazoprevir, ledipasvir/sofosbuvir, and ombitasvir/paritaprevir/ritonavir plus dasabuvir.[1-3] For these 3 DAAs, we required 8 weeks of treatment for patients without cirrhosis and 12 weeks for those with cirrhosis. For other DAAs, we applied 12 weeks to patients with and without cirrhosis. If patients filled prescriptions for more than 12 weeks (ie, patients were on regimens with expected duration >12 weeks), we applied 16 weeks for elbasvir/grazoprevir and 24 weeks for all other DAAs based on each drug's package insert. We considered an interval between fills of fewer than 60 days as continuation of the therapy.

eReferences

1. Vierling JM, Kugelmas M, Lawitz E, et al. Efficacy of an eight-week regimen of grazoprevir plus elbasvir with and without ribavirin in treatment-naive, noncirrhotic HCV genotype 1B infection. *J Hepatol*. 2015;62(suppl2):S618. doi: 10.1016/S0168-8278(15)30972-7.
2. Kowdley KV, Gordon SC, Reddy KR, et al; ION-3 Investigators. Ledipasvir and sofosbuvir for 8 or 12 weeks for chronic HCV without cirrhosis. *N Engl J Med*. 2014;370(20):1879-1888. doi: 10.1056/NEJMoa1402355.
3. Welzel TM, Asselah T, Dumas EO, et al. Ombitasvir, paritaprevir, and ritonavir plus dasabuvir for 8 weeks in previously untreated patients with hepatitis C virus genotype 1b infection without cirrhosis (GARNET): a single-arm, open-label, phase 3b trial. *Lancet Gastroenterol Hepatol*. 2017;2(7):494-500. doi: 10.1016/S2468-1253(17)30071-7.

© 2020 Jung J et al. *JAMA Network Open*

eAppendix 3. Details of Regression Specification

We estimated the multivariate regression at the person-period level to examine the association of DAA treatment with follow-up medical costs. The regression model is written as:

$$Y_{it} = \alpha + b(\text{FOLLOW\_TIME})_i + \lambda(\text{DAA})_t + \boldsymbol{\theta}(\text{FOLLOW\_TIME*DAA})_{it} + n(\text{PATIENT\_RISK})_{it} + \varepsilon_{it} \quad (1)$$

where i denotes the patient and t is time at 6-month intervals. Time t equals 0 for the pre-treatment period; and 1 for 6 months, 2 for 6-12 months, 3 for 12-18 months, 4 for 18-24 months, and 5 for 24-30 months after the completion of treatment. Y_{it} is the study outcome (total medical cost or HCV/liver cost) for patient i at time t.

FOLLOW_TIME_t denotes five indicators that represent each follow-up period after the completion of treatment. These indicators control for a common time trend over the follow-up periods. DAA_i is an indicator variable that equals 1 if patient i completed DAA treatment and 0 otherwise. This variable controls for permanent differences between DAA users and DAA non-users, such as high-risk status of non-users prior to treatment.

The interaction terms between FOLLOW_TIME and DAA are of interest. The coefficients on those interaction terms, **θ**, measure the mean adjusted difference between DAA users and non-users in changes in the outcome (costs) from the pre-treatment to each follow-up periods (6 months, 6-12 months, 12-18 months, 18-24 months, and 24-30 months after the completion of treatment).

PATIENT_RISK_{it} denotes the following characteristics for patient i at time t: age, gender, race, HIV/AIDS infection, having cancer, having substance use disorder, having kidney diseases, having hypertension, having diabetes, having cardiovascular diseases, having arthritis, having lung diseases, having anemia, and having eye diseases. ε_{it} are error terms, we clustered

- Estimation

We estimated this regression in a propensity-score matched sample (based on the nearest neighbor one-to-one matching with 0.1 caliper). We used a Generalized Linear Model (GLM) with a log link and the gamma distribution to account for the skewed distribution of the cost variables (the outcomes).* We estimated the model separately for patients with and without cirrhosis to allow heterogeneity in the associations of DAA use and all covariates with follow-up costs between those two groups.

- Calculation of cumulative cost reduction

The unit of analysis was a person-period (at 6-month intervals). The analysis allowed the number of follow-up periods to differ by patient because patients were followed up from DAA treatment to the end of study or death. For example, patients who completed DAA therapy in early 2015 were followed up 30 months (the maximum), while those with DAA treatment in 2016 were followed up 12 months. With this unbalanced data structure, it was not necessary to impute zero for those with fewer than 30 months follow-up, which would artificially inflate or deflate costs. We calculated cumulative effects from the regression results as the sum of cost reductions over all 6-month follow-up periods (i.e., the sum of the coefficients on the interaction terms, **θ**).

©2020 Jung J et al. *JAMA Network Open*

eFigure. Index Date and Definition of Study Periods

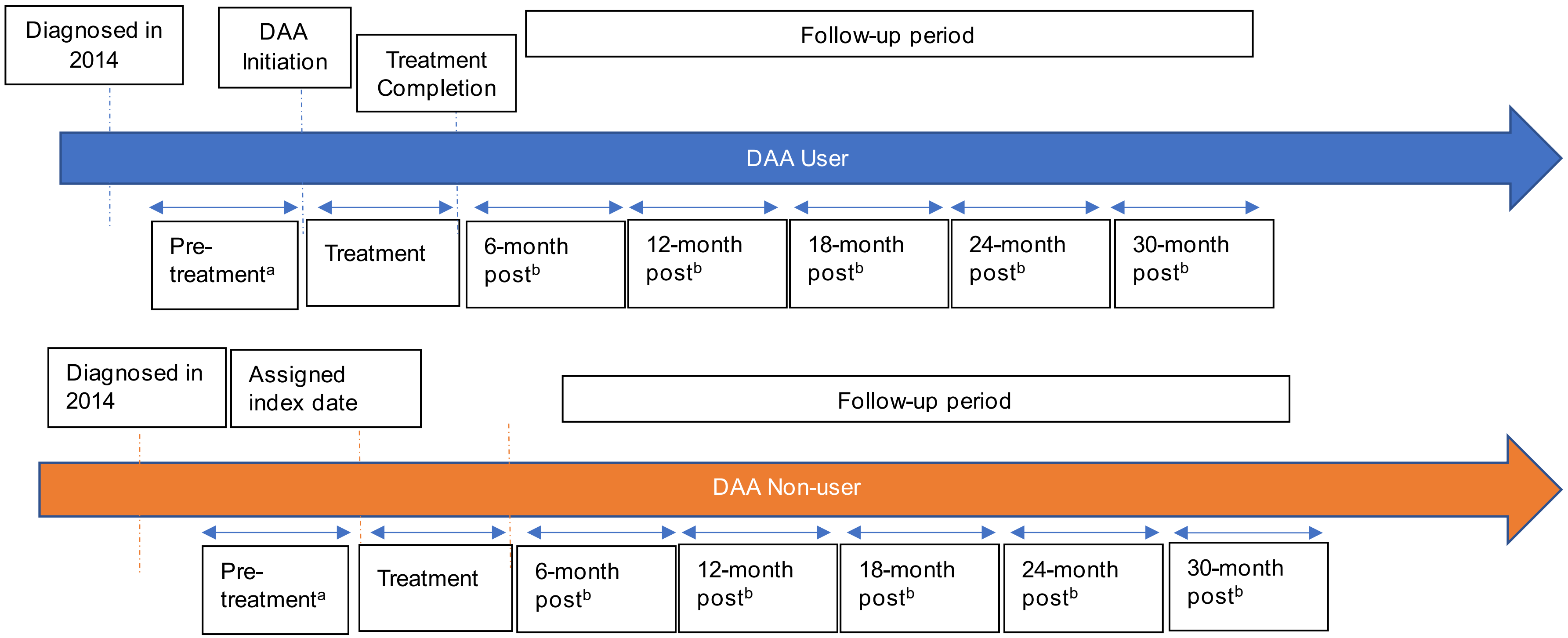

Abbreviations: DAA, Direct-acting antiviral drug;
[a] Pre-treatment among users refers to the 6-month period prior to initiation of DAAs among users, and among non-users it refers to the 6-month period prior to the assigned index date.
[b] Post refers periods after completion of DAA therapy among users, and among non-users it refers to the period after hypothetical treatment.

©2020 Jung J et al. *JAMA Network Open*

eTable 1. Definition and Data Sources of the Covariates and Outcomes Used in the Study

	Definition	Data Source
**Patient demographics**		
Age	Binary variables that indicate whether a patient's age was less than 65, 65-69,70-74, and 75 or above	Medicare MBSF file
Gender	Binary indicator that indicates whether a patient was female	Medicare MBSF file
Race	Binary variables that indicates whether a patient was White, African American, Hispanic, or other/unknown race.	Medicare MBSF file
**Health Risks**		
Cirrhosis	Binary indicator that indicates whether a patient had cirrhosis	Constructed by applying the standard CCW algorithm criteria (1 inpatient visit or 2 outpatient/carrier visits) to Medicare Part A and Part B claims file (ICD9 DX codes - 571.5, ICD10 DX codes -K74.0, K74.60, K74.69)
Decompensated cirrhosis	Binary indicator that indicates whether a patient had decompensated cirrhosis	If patient had an indicator for cirrhosis (created above) and atleast one claim for liver-related symptoms in Medicare Part A or Part B claims file (ICD9 DX codes -789.51, 789.59, 572.2, 572.3, 782.4, 567.23, 572.4, ICD10 DX codes -R18.0, R18.8, K72.90, K72.91, K76.6 ,R17, K65.2, K76.7)
Compensated cirrhosis	Binary indicator that indicates whether a patient had compensated cirrhosis	If patient had an indicator for cirrhosis (created above) and no claims for liver-related symptoms in Medicare Part A or Part B claims file (ICD9 DX codes -789.51, 789.59, 572.2, 572.3, 782.4, 567.23, 572.4, ICD10 DX codes -R18.0, R18.8, K72.90, K72.91, K76.6 ,R17, K65.2, K76.7)
HIV/AIDS	Binary indicator of whether the patient had HIV/AIDS	Medicare MBSF file - other chronic conditions[a]

© 2020 Jung J et al. *JAMA Network Open*

	Definition	Data Source
Hepatocellular cancer	Binary indicator of whether the patient had Liver/Hepatocellular cancer	Constructed by applying the standard CCW algorithm criteria (1 inpatient visit or 2 outpatient/carrier visits) to Medicare Part A and Part B claims file (ICD9 DX codes - 155.0, ICD10 DX codes -C22.0)
Anemia	Binary indicator of whether the patient had anemia	Medicare MBSF file - CCW Chronic conditions[a]
Lung Disease	Binary indicator of whether the patient had lung disease	Combining Asthma and COPD indicators available in Medicare MBSF file - CCW Chronic conditions[a]
Cancer	Binary indicator of whether the patient had cancer	Combining breast, lung, colon, prostate, hyperplasia indicators available in Medicare MBSF file - CCW Chronic conditions[a]
Cardiac disease	Binary indicator of whether the patient had cardiac disease	Combining lipedema, hypertension, atrial fibrillation, ischemic heart disease, heart failure, acute myocardial infarction, and stroke indicators available in Medicare MBSF file- CCW Chronic conditions[a]
Dementia	Binary indicator of whether the patient had dementia	Medicare MBSF file - CCW Chronic conditions[a]
Psychiatric conditions	Binary indicator of whether the patient had psychiatric conditions	Combining depression, anxiety, and bipolar indicators available in Medicare MBSF file- CCW Chronic conditions and other chronic conditions file[a]
Diabetes	Binary indicator of whether the patient had diabetes	Medicare MBSF file - CCW Chronic conditions[a]
Eye disease	Binary indicator of whether the patient had eye disease	Combining glaucoma and cataract indicators available in Medicare MBSF file- CCW Chronic conditions[a]
Kidney disorders	Binary indicator of whether the patient had kidney disorders	Medicare MBSF file - CCW Chronic conditions [a]
Drug and alcohol related disorder	Binary indicator of whether the patient had drug or alcohol related disorder	Combining alcohol use disorders and drug use disorders indicators available in Medicare MBSF file- other chronic conditions file[a]
Pre-treatment spending	Inflation-adjusted all-cause Medicare allowed payments[b] made during 6 months prior to index date.	Medicare provider analysis and review (MedPAR) file containing inpatient hospital and SNF claims, Medicare Hospital Outpatient file and Carrier file.

©2020 Jung J et al. *JAMA Network Open*

	**Definition**	**Data Source**
**Outcomes**<br>Hepatitis C related costs	Inflation-adjusted, hepatitis C related, Medicare allowed payments[b] made during each 6-month following the completion of treatment. The ICD codes used to identify hepatitis C related visits are provided in eAppendix 1.	Medicare provider analysis and review (MedPAR) file containing inpatient hospital and SNF claims, and Medicare Hospital Outpatient file and Carrier file.
Total medical costs	Inflation-adjusted all-cause Medicare allowed payments[b] made during each 6-month following the completion of treatment	Medicare provider analysis and review (MedPAR) file containing inpatient hospital and SNF claims, and Medicare Hospital Outpatient file and Carrier file.

Abbreviations: AIDS, Acquired immunodeficiency syndrome; CCW, CMS chronic conditions data warehouse; COPD, Chronic obstructive pulmonary disease; ESRD, End-stage renal disease; HIV, Human immunodeficiency virus; MBSF, Master beneficiary summary file; SNF, Skilled nursing facility;

[a] The diagnosis codes used by Medicare to identify these conditions are available at https://www2.ccwdata.org/web/guest/condition-categories

[b]The allowed payments included both Medicare reimbursements, patient responsibilities, and third party payments. In the MedPAR file, the allowed payment was calculated as the sum of all beneficiary payments including deductibles and coinsurance, and Medicare payments made for the claim, and any payment made by third party payers. The allowed payments were calculated similarly in the Medicare hospital outpatient file. In the carrier file, a single variable for allowed payments was available.

©2020 Jung J et al. *JAMA Network Open*

eTable 2. Results From Logit Regression for Propensity Score (Cirrhosis Group)[a]

	**Users that initiated DAAs 30-36 months after diagnosis**		**Users that initiated DAAs 24-30 months after diagnosis**		**Users that initiated DAAs 18-24 months after diagnosis**		**Users that initiated DAAs 12-18 months after diagnosis**		**Users that initiated DAAs 6-12 months after diagnosis**	
	N=1,024		N=1,734		N=1,865		N=1,979		N=2,380	
**Variables**	Coeff.	*P* Value	Coeff.	*P* Value	Coeff.	*P* Value	Coeff.	*P* Value	Coeff.	*P* Value
Age 65-70	-0.10	0.79	0.35	0.05	-0.04	0.83	0.24	0.11	0.33	0.02
Age 70-75	-0.76	0.14	-0.16	0.49	-0.44	0.05	-0.35	0.08	-0.33	0.09
Age >75	0.27	0.54	-0.16	0.55	-0.47	0.05	-0.45	0.05	-1.14	<0.001
Female	-0.23	0.44	-0.36	0.02	-0.13	0.38	-0.39	<0.001	-0.08	0.47
African American	0.63	0.09	0.56	<0.001	0.77	<0.001	0.76	<0.001	0.63	<0.001
Hispanic	-0.65	0.39	-0.45	0.23	-0.62	0.11	-0.90	0.01	-0.32	0.17
Other	-0.68	0.36	-0.28	0.41	0.21	0.45	-0.01	0.97	-0.11	0.65
Decompensated Cirrhosis	0.44	0.14	-0.12	0.45	-0.25	0.10	-0.12	0.34	-0.17	0.14
HIV/AIDS	-1.23	0.24	-0.33	0.35	-0.48	0.20	-0.34	0.29	-0.32	0.26
Hepatocellular cancer	0.05	0.93	0.27	0.31	-0.81	0.01	-0.11	0.61	-0.49	0.03
Anemia	-0.96	<0.001	-0.44	0.01	-0.20	0.19	-0.56	<0.001	-0.75	<0.001
Lung Disease	-0.21	0.49	-0.35	0.03	-0.27	0.08	-0.36	0.01	-0.59	<0.001
Cancer	-0.05	0.90	0.17	0.38	0.20	0.29	-0.50	0.01	-0.13	0.45
Cardiac disease	0.70	0.11	0.06	0.77	0.18	0.33	-0.09	0.56	-0.20	0.16
Dementia	-1.77	0.02	-0.53	0.03	-0.81	<0.001	-1.08	<0.001	-1.14	<0.001
Psychiatric conditions	0.02	0.94	0.30	0.06	-0.22	0.14	-0.22	0.08	-0.08	0.49
Diabetes	0.26	0.36	-0.04	0.81	-0.22	0.14	-0.18	0.15	-0.30	0.01
Eye disease	-0.31	0.43	-0.16	0.40	-0.10	0.58	-0.13	0.39	0.11	0.43
Kidney disorders	-0.21	0.50	-0.56	<0.001	-0.47	<0.001	-0.36	0.01	-0.71	<0.001
Drug and alcohol related disorder	-0.22	0.49	-0.24	0.13	-0.49	<0.001	-0.68	<0.001	-1.09	<0.001
Bone disease	0.04	0.87	-0.05	0.73	0.02	0.91	-0.08	0.51	-0.03	0.79
ESRD	-0.37	0.62	0.93	<0.001	-0.29	0.43	-1.30	<0.001	-1.50	<0.001
Prior 6-month costs	0.00	0.66	0.00	0.57	0.00	0.54	0.00	0.94	0.00	0.03

Abbreviations: AIDS, Acquired immunodeficiency syndrome; Coeff, Coefficient; DAA, Direct-acting antiviral drug; ESRD, End-stage renal disease; HIV, Human immunodeficiency virus;

[a] Propensity scores were calculated using users in each of the initiation categories and non-users that could be followed up for at least one 6-month follow-up period after the hypothetical treatment date assigned using prescription-time matching in each initiation category

© 2020 Jung J et al. *JAMA Network Open*

eTable 3. Results From Logit Regression for Propensity Score (Non-Cirrhosis Group)[a]

	Users that initiated DAAs 30-36 months after diagnosis		Users that initiated DAAs 24-30 months after diagnosis		Users that initiated DAAs 18-24 months after diagnosis		Users that initiated DAAs 12-18 months after diagnosis		Users that initiated DAAs 6-12 months after diagnosis	
	N=12,060		N=19,916		N=21,359		N=22,272		N=23,487	
**Variables**	Coeff.	*P* Value	Coeff.	*P* Value	Coeff.	*P* Value	Coeff.	*P* Value	Coeff.	*P* Value
Age 65-70	-0.02	0.93	0.13	0.24	0.26	<0.001	0.26	<0.001	0.29	<0.001
Age 70-75	0.03	0.88	-0.16	0.25	-0.18	0.13	-0.13	0.18	-0.24	0.01
Age >75	-0.54	0.05	-0.88	<0.001	-0.65	<0.001	-0.83	<0.001	-1.08	<0.001
Female	-0.09	0.53	-0.13	0.11	-0.03	0.67	-0.01	0.90	-0.06	0.21
African American	0.53	<0.001	0.43	<0.001	0.37	<0.001	0.47	<0.001	0.47	<0.001
Hispanic	-0.72	0.16	0.40	0.04	-0.19	0.34	-0.21	0.22	-0.18	0.20
Other	-0.21	0.52	0.02	0.90	-0.40	0.03	-0.43	<0.001	-0.31	0.01
Decompensated Cirrhosis	0.42	0.07	0.35	0.02	0.59	<0.001	0.50	<0.001	0.22	0.02
HIV/AIDS	1.17	0.06	0.66	0.21	0.46	0.28	0.38	0.29	-0.06	0.86
Hepatocellular cancer	-0.22	0.18	-0.10	0.33	-0.01	0.94	-0.01	0.92	-0.09	0.11
Anemia	-0.19	0.23	0.04	0.69	-0.14	0.07	-0.27	<0.001	-0.39	<0.001
Lung Disease	0.13	0.52	0.10	0.40	0.16	0.12	-0.12	0.20	0.05	0.53
Cancer	0.10	0.56	0.11	0.25	-0.11	0.17	0.14	0.03	-0.05	0.34
Cardiac disease	-0.64	0.01	-0.35	0.02	-0.76	<0.001	-0.77	<0.001	-1.12	<0.001
Dementia	-0.04	0.78	-0.02	0.86	-0.16	0.02	-0.16	0.01	-0.15	<0.001
Psychiatric conditions	-0.01	0.96	-0.21	0.03	0.02	0.77	-0.11	0.09	-0.11	0.04
Diabetes	0.25	0.16	0.10	0.36	0.10	0.26	0.27	<0.001	0.29	<0.001
Eye disease	0.51	<0.001	-0.12	0.25	-0.18	0.04	-0.38	<0.001	-0.53	<0.001
Kidney disorders	0.00	0.98	-0.12	0.16	-0.16	0.02	-0.40	<0.001	-0.55	<0.001
Drug and alcohol related disorder	0.07	0.63	0.18	0.03	0.10	0.13	-0.06	0.29	0.01	0.83
Bone disease	-0.03	0.90	0.33	0.06	-0.04	0.79	-0.99	<0.001	-1.45	<0.001
ESRD	0.00	0.67	0.00	0.78	0.00	0.75	0.00	0.51	0.00	0.78
Prior 6-month costs	-0.02	0.93	0.13	0.24	0.26	<0.001	0.26	<0.001	0.29	<0.001

Abbreviations: AIDS, Acquired immunodeficiency syndrome; Coeff, Coefficient; DAA, Direct-acting antiviral drug; ESRD, End-stage renal disease; HIV, Human immunodeficiency virus;

[a] Propensity scores were calculated using users in each of the initiation categories and non-users that could be followed up for at least one 6-month follow-up period after the hypothetical treatment date assigned using prescription-time matching in each initiation category

eTable 4. Patient Characteristics of Matched and Unmatched DAA Non-Users

	Cirrhosis group			Non-cirrhosis group		
	Unmatched DAA non-users (N=2,844)	Matched DAA non-users (N=1,675)	*P* value[b]	Unmatched DAA non-users (N=23,685)	Matched DAA non-users (N=5,924)	*P* value[b]
**Variable**	No. (%)	No. (%)		No. (%)	No. (%)	
Mean Age (SD)	62.8 (10.7)	62.1 (10.2)	0.04	59.2 (12.9)	59.4 (11.7)	0.34
**Age**						
Age <65	1,629 (57.3%)	972 (58.0%)		1,574 (66.4%)	3,714 (62.7%)	
Age 65-70	496 (17.4%)	364 (21.7%)		3,336 (14.1%)	1,319 (22.3%)	
Age 70-75	316 (11.1%)	186 (11.1%)		1,890 (8.0%)	551 (9.3%)	
Age >75	403 (14.2%)	153 (9.1%)	<0.001	2,754 (11.6%)	340 (5.7%)	<0.001
**Gender**						
Female	1,049 (36.9%)	611 (36.5%)		10,297 (43.5%)	2,470 (41.7%)	
Male	1,795 (63.2%)	1,064 (63.5%)	0.78	13,388 (56.5%)	3,454 (58.3%)	0.01
**Race**						
White	2022 (71.1%)	1,217 (72.7%)		16,486 (69.6%)	3,909 (66.0%)	
African American	482 (16.9%)	311 (18.6%)		5,174 (21.8%)	1,634 (27.6%)	
Hispanic	168 (5.9%)	61 (3.6%)		811 (3.4%)	164 (2.8%)	
Other	162 (6.1%)	86 (5.1%)	0.003	1,214 (5.1%)	217 (3.7%)	<0.001
**Cirrhosis Type**						
Decompensated cirrhosis	1,350 (47.5%)	518 (30.9%)		-	-	
Compensated cirrhosis	1,494 (52.5%)	1,157 (69.1%)	<0.001	-	-	
**Conditions**						
HIV/AIDS	98 (3.5%)	58 (3.5%)	0.98	1,121 (4.7%)	466 (7.9%)	<0.001
Hepatocellular cancer	245 (8.6%)	137 (8.2%)	0.61	147 (0.6%)	37 (0.6%)	0.97
Anemia	2,136 (75.1%)	696 (41.6%)	<0.001	10,055 (42.4%)	1,624 (27.4%)	<0.001
Lung Disease	1,322 (46.5%)	499 (29.8%)	<0.001	9,289 (39.2%)	1,511 (25.5%)	<0.001
Cancer	522 (18.4%)	207 (12.4%)	<0.001	3,259 (13.8%)	701 (11.8%)	<0.001
Cardiac disease	2,539 (89.3%)	1,324 (79.0%)	<0.001	18,600 (78.5%)	4,172 (70.4%)	<0.001
Dementia	497 (17.5%)	87 (5.2%)	<0.001	2,741 (11.6%)	272 (4.6%)	<0.001
Psychiatric conditions	1,688 (59.3%)	858 (51.2%)	<0.001	14,577 (61.6%)	3,124 (52.7%)	<0.001
Diabetes	1,512 (53.2%)	689 (41.1%)	<0.001	8,536 (36.0%)	1,817 (30.7%)	<0.001
Eye disease	464 (16.3%)	304 (18.2%)	0.11	3,445 (14.6%)	1,051 (17.7%)	<0.001
Kidney disorders	1,531 (53.8%)	557 (33.2%)	<0.001	7,631 (32.2%)	1,395 (23.6%)	<0.001
Drug and alcohol related disorder	1,927 (67.8%)	946 (56.5%)	<0.001	14,293 (60.3%)	2,917 (49.2%)	<0.001
Bone disease	1,226 (43.1%)	664 (39.6%)	0.02	10,007 (42.2%)	2,549 (43.0%)	0.28
ESRD	359 (12.6%)	69 (4.1%)	<0.001	1,987 (8.4%)	242 (4.1%)	<0.001

Abbreviations: AIDS, Acquired immunodeficiency syndrome; DAA, Direct-acting antiviral drug; ESRD, End-stage renal disease; HIV, Human immunodeficiency virus;

[a] P values were calculated with unpaired, 2-tailed t tests for binary and continuous variables and $\chi 2$ tests for categorical variables.

©2020 Jung J et al. *JAMA Network Open*

eTable 5. Unadjusted Spending Over Time and Death Rates Between DAA Users and Non-Users

	Pre-treatment[a]	6 months post[b]	12 months post[b]	18 months post[b]	24 months post[b]	30 months post[b]
**Cirrhosis Group**						
DAA Users (N)	1,675	1,675	1,397	1,015	625	118
DAA Non-users (N)	1,675	1,675	1,288	835	431	164
HCV/Liver-related costs (Mean, SD)						
DAA Users	$3,422(12,155)	$2,511(11,002)	$1,872(9,462)	$1,672(6,601)	$1,910(8,602)	$1,362(5,448)
DAA Non-users	$2,328(7,449)	$2,955(11,218)	$2,587(11,398)	$2,533(10,176)	$1,953(7,237)	$1,425(6,687)
Total medical costs (Mean, SD)						
DAA Users	$12,665(28,126)	$11,086(21,675)	$9,890(21,265)	$8,894(18,615)	$8,929(19,480)	$9,389(15,987)
DAA Non-users	$14,666(25,743)	$16,617(33,572)	$15,200(36,376)	$13,135(25,098)	$13,714(23,747)	$10,782(25,048)
Deaths (N, % among those at risk)						
DAA Users			35(2.1%)	41(2.9%)	36(3.6%)	8(1.3%)
DAA Non-users			120(7.2%)	82(6.4%)	32(3.8%)	12(2.8%)
**Non-Cirrhosis Group**						
DAA Users (N)	5,924	5,924	5,014	3,869	2,375	519
DAA Non-users (N)	5,924	5,924	4,898	3,536	2,012	579
HCV/Liver-related costs (Mean, SD)						
DAA Users	$879(4,336)	$311(2,024)	$230(2,255)	$170(1,246)	$173(1,420)	$136(794)
DAA Non-users	$260(2,691)	$289(3,989)	$255(3,393)	$237(1,952)	$220(3,048)	$163(999)
Total medical costs (Mean, SD)						
DAA Users	$7,911(20,074)	$7,881(24,769)	$7,425(22,483)	$7,361(26,036)	$6,949(22,170)	$7,893(40,140)
DAA Non-users	$10,614(27,715)	$11,406(36,713)	$10,605(33,475)	$10,461(42,222)	$10,679(62,014)	$10,159(38,098)
Deaths (N, % among those at risk)						
DAA Users			37(0.6%)	43(0.9%)	43(1.1%)	11(0.5%)
DAA Non-users			121(2.0%)	127(2.6%)	61(1.7%)	20(1.0%)

	6-month pre-treatment		6-month treatment		Average 6-month post-treatment		Drug Costs
	**HCV/Liver costs**	**Non-HCV/Liver costs**	**HCV/Liver costs**	**Non-HCV/Liver costs**	**HCV/Liver costs**	**Non-HCV/Liver costs**	
	Mean (SD)	Mean (SD)	Mean (SD)	Mean (SD)	Mean (SD)	Mean (SD)	Mean (SD)
**Cirrhosis Group**							
DAA Incomplete	$6,398(36,660)	$10,403(17,653)	$3,554(8,039)	$8,756(19,610)	$2,830(9,219)	$9,922(15,304)	$82,973(40,994)
DAA Users	$3,422(12,155)	$9,242(22,741)	$2,186(5,838)	$7,755(21,711)	$2,337(9,380)	$8,709(15,093)	$102 634(44,184)
DAA Non-users	$2,328(7,449)	$12,338(24,060)	$2,511(11,002)	$12,974(27,870)	$2,974(9,008)	$13,680(27,654)	-
**Non-Cirrhosis Group**							
DAA Incomplete	$884(1,766)	$8,625(21,495)	$866(3,474)	$10,288(23,800)	$621(969)	$9,312(16,933)	$71,768(41,852)
DAA Users	$879(4,336)	$7,033(19,339)	$620(1,745)	$6,560(19,782)	$269(1,613)	$7,830(22,401)	$80,898(27,526)

©2020 Jung J et al. *JAMA Network Open*

DAA Non-users	$260(2,691)	$10,354(27,493)	$311(2,024)	$10,075(39,755)	$279(2,761)	$11,314(34,040)	-

Abbreviations: DAA, Direct-acting antiviral drug; HCV, Hepatitis C virus; SD, Standard deviation;

[a] Pre-treatment among users refers to the 6-month period prior to initiation of DAAs among users, and among non-users it refers to the 6-month period prior to the assigned index date.

[b] Post refers periods after completion of DAA therapy among users, and among non-users it refers to the period after hypothetical treatment.

eTable 6. Results from Regression With Patients That Survived During Follow-Up[a]

	Among cirrhosis group (N=11,267)		Among non-cirrhosis group (N=44,813)	
	Users vs. Non-Users	*P* Value	Users vs. Non-Users	*P* Value
	Differences (95% CI)		Differences (95% CI)	
**HCV/Liver-related costs ($)**				
Time after treatment				
6-months	-2,039 (-2,783 to -1,296)	<0.001	-432 (-533 to -331)	<0.001
12-months	-1,800 (-2,601 to -1,000)	<0.001	-547 (-656 to -438)	<0.001
18-months	-1,489 (-2,358 to -621)	0.001	-510 (-612 to -407)	<0.001
24-months	-346 (-1,567 to 874)	0.58	-401 (-497 to -305)	<0.001
30-months	-1,388 (-2,520 to -257)	0.02	-351 (-458 to -244)	<0.001
**Total medical costs ($)**				
Time after treatment				
6-months	-2,438 (-4,335 to -540)	0.01	-1,121 (-2,117 to -124)	0.03
12-months	-995 (-2,826 to 836)	0.29	-1,620 (-2,650 to -589)	0.002
18-months	244 (-1,874 to 2,362)	0.82	-1,873 (-4,042 to 296)	0.09
24-months	-924 (-4,290 to 2,442)	0.59	-1,350 (-3,111 to 411)	0.13
30-months	1,235 (-3,198 to 5,669)	0.59	-827 (-4,249 to 2,595)	0.64

Abbreviations: CI, Confidence intervals; DAA, Direct-acting antiviral drug; HCV, Hepatitis C virus;

[a] Multivariate regression was used to examine the association of DAA treatment with spending outcomes in patients that survived during follow-up period. Changes in medical costs were compared between DAA users and non-users over 30 months of follow-up after the completion of DAA treatment using a difference-in-differences approach. eAppendix 3 provides further details of the regression model and describes the variables used in the model.

© 2020 Jung J et al. *JAMA Network Open*

eTable 7. Unadjusted Spending Among DAA Users by Time-to-Treatment

	6 months after diagnosis	Pre-treatment[a]	6 months post[b]	12 months post[b]	18 months post[b]	24 months post[b]
**Cirrhosis Group**						
Use <= 1yr of diagnosis (N)	598	598	598	564	537	433
Use > 1yr of diagnosis (N)	1,077	1,077	1,077	833	478	192
HCV/Liver-related costs (Mean, SD)						
Use <= 1yr of diagnosis	$3,344 (8,582)	$3,042 (8,029)	$2,810 (11,587)	$1,851 (9,888)	$1,476 (6,571)	$1,877 (9,380)
Use > 1yr of diagnosis	$2,083 (8,034)	$3,634 (13,927)	$2,345 (10,665)	$1,886 (9,169)	$1,893 (6,634)	$1,985 (6,536)
Total medical costs (Mean, SD)						
Use <= 1yr of diagnosis	$12,008 (20,736)	$9,970 (19,528)	$10,017 (21,009)	$8,778 (18,991)	$8,319 (15,642)	$9,223 (21,376)
Use > 1yr of diagnosis	$12,980 (23,849)	$14,161 (31,826)	$11,680 (22,023)	$10,643 (22,655)	$9,539 (21,468)	$8,265 (14,339)
**Non-Cirrhosis Group**						
Use <= 1yr of diagnosis (N)	2,326	2,326	2,326	2,247	2,161	1,802
Use > 1yr of diagnosis (N)	3,598	3,598	3,598	2,767	1,708	573
HCV/Liver-related costs (Mean, SD)						
Use <= 1yr of diagnosis	$909 (2,202)	$824 (1,561)	$280 (1,481)	$248 (2,800)	$170 (1,218)	$185 (1,542)
Use > 1yr of diagnosis	$677 (2,695)	$914 (542)	$332 (2,308)	$215 (1,689)	$171 (1,280)	$135 (937)
Total medical costs (Mean, SD)						
Use <= 1yr of diagnosis	$9,145 (28,162)	$7,073 (21,987)	$6,537 (19,968)	$6,781 (20,430)	$7,088 (25,873)	$7,202 (24,594)
Use > 1yr of diagnosis	$11,487 (28,737)	$8,453 (18,717)	$8,751 (27,396)	$7,948 (24,013)	$7,708 (26,244)	$6,152 (11,603)

Abbreviations: DAA, Direct-acting antiviral drug; HCV, Hepatitis C virus; SD, Standard deviation;
[a] Pre-treatment refers to the 6-month period prior to initiation of DAAs among users.
[b] Post refers periods after completion of DAA therapy among users.

© 2020 Jung J et al. *JAMA Network Open*

eTable 8. Adjusted Outcomes Associated With DAA Use by Time to Treatment from HCV Diagnosis (by Cirrhosis)[a]

	Among cirrhosis group (N=12,573)		Among non-cirrhosis group (N=46,498)	
	Users vs. Non-Users	*P* Value	Users vs. Non-Users	*P* Value
	Differences (95% CI)		Differences (95% CI)	
**HCV/Liver-related costs ($)**				
6-months[b]				
Use <=1yr of diagnosis	-2,459 (-3,596 to -1,321)	<0.001	-520 (-656 to -385)	<0.001
Use > 1yr of diagnosis	-2,558 (-3,525 to -1,590)	<0.001	-480 (-606 to -355)	<0.001
12-months				
Use <=1yr of diagnosis	-2,632 (-3,800 to -1,465)	<0.001	-667 (-813 to -522)	<0.001
Use > 1yr of diagnosis	-2,234 (-3,306 to -1,163)	<0.001	-587 (-722 to -452)	<0.001
18-months				
Use <=1yr of diagnosis	-2,237 (-3,380 to -1,094)	<0.001	-577 (-711 to -542)	<0.001
Use > 1yr of diagnosis	-1,387 (-2,558 to -216)	0.02	-574 (-701 to -446)	<0.001
24-months				
Use <=1yr of diagnosis	-135 (-2,185 to 1,915)	0.90	-458 (-590 to -327)	<0.001
Use > 1yr of diagnosis	-1,298 (-2,343 to -253)	0.02	-499 (-625 to -374)	<0.001
30-months				
Use <=1yr of diagnosis	-1,689 (-3,110 to -268)	0.02	-396 (-528 to -264)	<0.001
**Total medical costs ($)**				
6-months				
Use <=1yr of diagnosis	-2,718 (-5,333 to -102)	0.04	-1,852 (-3,086 to -618)	<0.001
Use > 1yr of diagnosis	-2,959 (-5,037 to -882)	0.01	-905 (-1,984 to 174)	0.10
12-months				
Use <=1yr of diagnosis	-1,967 (-4,564 to 628)	0.14	-2,288 (-3,515 to -1,060)	<0.001
Use > 1yr of diagnosis	-2,135 (-4,283 to 12)	0.05	-1,181 (-2,354 to -8)	0.05
18-months				
Use <=1yr of diagnosis	499 (-2,387 to 3,385)	0.74	-2,381 (-4,625 to -138)	0.04
Use > 1yr of diagnosis	-108 (-2,302 to 2,085)	0.92	-1,340 (-3,694 to 1,014)	0.26
24-months				
Use <=1yr of diagnosis	-193 (-4,303 to 3,917)	0.93	-1,541 (-3,439 to 358)	0.11
Use > 1yr of diagnosis	-3,338 (-6,769 to 93)	0.06	-1,754 (-3,667 to 158)	0.07
30-months				
Use <=1yr of diagnosis	1,099 (-3,834 to 6,033)	0.66	-947 (-4,268 to 2,374)	0.58

Abbreviations: CI, Confidence intervals; DAA, Direct-acting antiviral drug; HCV, Hepatitis C virus;

[a] The DAA users were grouped into two categories by time-to-DAA initiation: within 12 months and after 12 months. Then, the changes in medical costs were compared between each of these two categories and non-users. The model controlled for common time trends, permanent differences across categories of DAA users, and patient characteristics.

[b] All periods refer to periods after completion of DAA therapy among users, and among non-users it refers to the period after hypothetical treatment.

© 2020 Jung J et al. *JAMA Network Open*
